# Impacts of florfenicol on immunity, antioxidant activity, and histopathology of *Oreochromis niloticus*: a potential protective effect of dietary *spirulina platensis*

**DOI:** 10.1007/s11259-023-10189-9

**Published:** 2023-08-11

**Authors:** Nagwa I.S. Abu-Zahra, Mohamed M. Elseify, Ayman A. Atia, Eman T. Al-sokary

**Affiliations:** 1 Department of Fish Diseases, Kafrelsheikh Provincial Lab, Animal Health Research Institute (AHRI) Agriculture Research Center (ARC), Giza, Egypt; 2 Department of Immunology, Kafrelsheikh Provincial Lab, Animal Health Research Institute (AHRI) Agriculture Research Center (ARC), Giza, Egypt; 3 Department of Pathology, Kafrelsheikh Provincial Lab, Animal Health Research Institute (AHRI) Agriculture Research Center (ARC), Giza, Egypt; 4 Department of Biochemistry, Kafrelsheikh Provincial Lab, Animal Health Research Institute (AHRI) Agriculture Research Center (ARC), Giza, Egypt

**Keywords:** *Oreochromis niloticus*, Florfenicol, *Spirulina platensis*, Antioxidants, Immune parameters

## Abstract

The misuse of antibiotics enhances the development of resistant microorganisms and decreases the efficacy of treatments. Florfenicol (FF) is one of the antibiotics approved for use in aquaculture in Egypt. Because of its extensive usage, potential negative impacts on aquatic creatures are a major concern. This motivates us to search for an appropriate neoadjuvant to work synergistically with FF and reduce adverse effects. Results from this study will contribute towards improving the understanding of the impacts of FF on *Oreochromis niloticus* and the possible amelioratory effects of *Spirulina platensis* algae (SP). *O. niloticus* (n = 240; 40 ± 2.5 g) were fed on two diets supplemented with or without SP for 4 weeks, then divided into four treatments each in three replicates (n = 60/treatment). G1; was fed a control diet, and the other groups were fed diets supplemented with FF (10 mg /kg of BW, G2), SP (2 g/kg of diet, G3), or FF + SP (G4) for 10 days. Among the four groups, the SP group (G3) had the best immunostimulatory effects as observed by a significant (p < 0.05) elevation in phagocytic activity, phagocytic index, IL6, and TNF-α. The treatment with FF had significantly impacted hepatic and renal tissues, as the values of liver enzymes and creatinine demonstrated tissue deterioration and also resulted in oxidative stress, which was expressed by an increase of GPx, CAT, and SOD in (G2). Additionally, the combined FF + SP improved the hematological parameters and decreased the oxidative damage induced by FF (G4). Thus, it was clear that FF has harmful effects on *O. niloticus* and that SP can modulate such impacts. These data recommend the use of SP as an effective immunostimulant and a probable adjuvant to FF in *O. niloticus* diets to attain maximum disease resistance.

## Introduction

Conservation and enhancement of fish health are the primary driving forces behind aquaculture. Aquaculture productivity has increased due to the rise in demand for fish and fish products. This increase is associated with stress conditions that provide a suitable environment for infectious microorganisms (Dehghan et al. 2016), resulting in a general rise in the incidence of disease outbreaks. As a result, the use of antimicrobial medicated feed has increased overall in the past few years. It is mostly used to treat bacterial disease epidemics (Henriksson et al. 2018). For example, in 2016, about 382,500 kg of antimicrobials were utilized globally by the industry of salmon, and oxytetracycline and florfenicol were the most commonly used antibiotics (Love et al. 2020). Besides the development of bacterial strains resistant to antibiotics, overuse of antibiotics can have detrimental effects on fish, the environment, and human health through the accumulation of antibiotic residues in tissues (Henriksson et al. 2018). Antibiotics are not recommended in aquaculture because of their residual impacts on the tissues of aquatic animals (Khajeali et al. 2012). There are conflicting findings regarding how antibiotics affect fish immunity; for example, some authors have described positive impacts (Ezatrahimi et al. 2019) or only negligible effects (Guardiola et al. 2012), and several studies have revealed adverse impacts. For instance, during a vaccination study, florfenicol lowered the oxidative burst activity and reduced the survival rate (Lundén et al. 1999), and several studies reported that oxytetracycline suppresses fish immunity (Rijkers et al. 1980; Tapia-Paniagua et al. 2015).

Florfenicol (FF) is a cheap, wide-ranging antimicrobial with a low toxicity level (Ezatrahimi et al. 2019). In addition to chloramphenicol’s properties, such as its broad-spectrum activities against both Gram + ve and -ve bacteria and fast tissue penetration, FF has a prolonged elimination half-life. Due to the addition of a hydroxyl group to the chemical structure of FF instead of a fluorine group, it is more resistant to enzymatic inactivation, making its effects broader spectrum to bacterial strains that are resistant to chloramphenicol (Anadón et al. 2008).

FF is broadly used in aquaculture for treating infections in several countries, including China, Brazil, the United States (Done et al. 2015), Egypt, Norway, South Korea, and Vietnam (Limbu et al. 2018). FF is the most recent antibiotic to be licensed by the US Food and Drug Administration (USFDA), joining oxytetracycline dihydrate, sulfamerazine, and sulfadimethoxine/ormetoprim (USFDA 2021). Earlier studies reported the effective antibacterial activity of FF against common fish bacterial species, including *Vibrio anguillarum* (Seljestokken et al. 2006), A. *salmonicida, A. hydrophila, Flavobacterium psychrophilum, Yersinia ruckeri* (Carraschi et al. 2011), and *Edwardsiella ictaluri* (Gaunt et al. 2015). The recommended doses for catfish and finfish in the US are 10–15 mg/kg body weight daily for ten days to control mortalities caused by *Edwardsiella ictaluri* (FARAD 2019; USFDA 2021). FF was permitted to be used in fish orally or intramuscularly in China at labeled dosages of 10–15 mg/kg and 5–10 mg/kg body weight daily for 3–5 successive days, respectively (CCVP 2015). Despite its increased use, FF is toxic in several research studies (Hu et al. 2014), and (EMA1996) reported alterations in hematologic markers, an increase in liver weight, in addition to testicular degeneration in rats and dogs following repetitive oral doses of FF. Dietary medication is the utmost commonly used way of drug administration in aquaculture. Furthermore, fishers include FF (10 mg/L) in the transport water to boost larval viability (Ren et al. 2017). In a toxicity trial on FF conducted with *O. niloticus* (Shiroma et al. 2020), the authors claimed that the 48-hour exposure duration was sufficient to cause oxidative stress, resulting in cellular oxidative damage. As a result, even at sublethal doses of about 1/100 LC50-48-hour, FF may be toxic for fish species. Bardhan et al. (2022a) demonstrated that FF caused oxidative stress and histopathological changes in *O. niloticus* suggesting its hepatotoxicity and nephrotoxicity. Therefore, it is critical to determine FF’s impacts on fish’s immune, antioxidant, and physiological levels and search for healthy and eco-friendly substitutes.

*Spirulina platensis* (SP), freshwater blue-green microalgae, seems to have a high nutritional content of vitamins and proteins (60–70%). Furthermore, it contains important fatty acids including palmitic, linoleic, and linolenic acids, as well as minerals (Habib et al. 2008). Bioremediation with microalgae is appealing due to its diverse biological, chemical, and nutritional features (Sayed et al. 2020). SP has been shown to improve growth performance, immune status, and disease resistance in a variety of fish species, including *O. niloticus*, *Clarias gariepinus*, and *Oryzias latipes* (Sayed et al. 2019). Earlier studies that used SP as feed additives for fish and crustaceans showed it to improve both specific and non-specific immunity in *O. niloticus* (Takeuchi et al. 2002), *Acipenser transmontanus* (Palmegiano et al. 2008), and *Clarias gariepinus* (Promya and Chitmanat 2011). The use of SP has been demonstrated to enhance resistance to *Vibrio alginolyticus* infection in *Litopenaeus vannamei* (Chen et al. 2016).

As far as we know, no earlier studies have evaluated the co-effect of SP and FF on fish, particularly *O. niloticus*. Thus, this study intended to evaluate the effects of FF and SP as feed additives on the blood biochemical parameters, antioxidant status, immunity, and disease susceptibility of *O. niloticus* and whether SP enhances the efficacy of FF treatment.

## Materials and methods

### Experimental fish and culture facilities

The study methodology, protocol, and animal care procedures all followed the required standards and regulations of the Institutional Aquatic Animal Care and Use Committee, Faculty of Aquatic and Fisheries Sciences, Kafrelsheikh University. *O. niloticus* fish (n = 240, 40 ± 2.5 g), were obtained from a local fish farm (Kafrelsheikh governorate, Egypt) and transmitted alive to the lab of the Animal Health Research Institute (AHRI), Kafrelsheikh province lab, to be used in the laboratory trial for treatment with FF and SP and artificial infection. Fish were adapted to the lab conditions and fed *ad libitum* for 2 weeks before the experiment. Then, fish were randomly distributed into 12 glass aquaria (50 **×** 40 **×** 40 cm, n = 20 fish/ aquarium). Two diets were formulated to contain SP at 0 or 2 g/kg diet. The control diet was prepared as indicated by (NRC 2011) (Table 1). In the presence of water and oil, all the ingredients were well mixed and then supplemented with SP (Fresh-Life Pharma, Canada).

**Table 1:** physical and chemical composition of the control diet

Physical composition		Chemical composition	
Components	%	Item	%
Fish meal (55%) Corn grain (8.5%) Corn gluten (60%) Wheat bran (13.8%) Soya bean meal (44%) soya oil Mineral and Vitamin mixture ^a^ Di- Calcium phosphate Salt Carboxy Meth. Cellulose DL. Meth Total	9 33.675 10 1.5 40 5 0.3 1.2 0.2 0.2 0.125 100	Crude protein (CP) Ash Crude fiber (CF) Methionine ^b^ Lysine ^b^ Calcium ^b^ Phosphorus ^b^ Digestible energy (DE) ^c^	31.6 5.61 5.14 0.74 1.7 0.74 0.6 3193 (KJ/kg diet)

^a^ Minerals mixture (mg/kg diet); FeC6H5O7.3H2O, 40; MnCl2.4H2O, 80; Cu (OAc) 2.H2O, 4; ZnCO3, 50; CaIO3.6H2O,0.5; CoCl3.6H2O, 0.2 and Na2SeO3, 0.2

Vitamin mixture ((IU or mg/kg diet)); Vit A 5000 IU, Vit D3 1000 IU, α − tocopherol acetate 20.1 g, menadione(k3) 2 g, thiamine (B1) 2 g, riboflavin (B2) 5 g, pyridoxine (B6) 1.4, cyanocobalamin (B12) 0.02 g, Pantothenic acid (B5) 10 g, Folic acid 0,75 g, Biotin 0.2 g, nicotinic acid 30 g

^b^ A formula based on the chemical content of feedstuff nutrients was used to estimate the amounts of Methionine, Lysine, Calcium, and Phosphorus (NRC 2011)

c Utilizing a formula based on the chemical content of the nutrients in feedstuffs, digestible energy was calculated (NRC 2011)

The 1st group (CTR; 6 aquaria) was fed the control diet, while the 2nd group (SP; 6 aquaria) was fed an SP-enriched diet (2 g/kg diet). Diets were delivered to fish groups twice per day (9:00 a.m.; 2:00 p.m.) at a rate of 3% BW for 4 weeks and feed intake was assessed visually following each feeding. Feed still in tanks one hour after each feeding time, was drained into a pre-weighed tank, left to dry overnight, collected into a container, and weighed daily. Water parameters were regularly checked and recorded 6.1 ± 0.4 mg/L for dissolved oxygen (using the Standard Polarographic DO Probe -HI76407-Hanna Instruments Inc., RI, USA) and 25 ± 2^o^C and 7.2 ± 0.2 for temperature and pH respectively (using the digital waterproof pHep®pH/temperature tester -HI98128-Hanna Instruments Inc., RI, USA). Daily siphoning of the aquariums was done and about half of the water was changed with dechlorinated water.

After that as shown in Table 2, fish were divided into 4 groups (n = 60 fish/group, each group had a triplicate of 20 fish) and 10 mg/kg BW of FF (Sigma–Aldrich Chemical, USA) was mixed with a coating solution (1% gelatin and 1% tamarind gum) and sprayed on half of the control and SP- supplemented diets. Fish continued feeding experimental diets for 10 consecutive days. The total feeding period on SP equals 38 days and on FF equals 10 days.

### Blood sampling

At the end of the feeding period (SP equals 38 days and FF equals 10 days), all fish were in good health showing no clinical signs, no mortalities (except two mortalities in G2), and actively feeding. All fish were starved for a full day before sampling, anesthetized via 150 mg/l MS222, and blood was collected from the caudal vessels into 100 IU/ml sodium heparin to evaluate the phagocytic assay and hematological indices which were done within 24 h from sampling. Based on the serum sample analysis, the other parameters were assessed. The serum was obtained by centrifuging the clotted blood at 4 °C for 4000 rpm/5 minutes, then collected in sterilized Eppendorf tubes and kept at -20 °C until analysis (done within 2 weeks from sampling).

### Treatment efficacy of FF and SP

At the end of the feeding trial, the therapeutic efficacy of FF and SP was evaluated through an experimental challenge of *O. niloticus* with a pathogenic strain of *A. hydrophila*, kindly provided by the fish diseases unit, Kafrelsheikh Provincial Lab, Animal Health Research Institute. The bacterial strain was earlier isolated from diseased *O. niloticus* with characteristic symptoms of MAS and it was identified using phenotypic and molecular techniques. The isolate was stored at -80 °C until use. According to El Latif et al. (2019), the bacterial suspension was prepared using McFarland standard tubes. After the feeding trial, 20 fish from each group were intraperitoneally injected with a 0.1 ml dose of 24 h *A. hydrophila* old broth culture (1 × 10^7^CFU/ ml) following Abu-Elala et al. (2015) as shown in Table 2. The infected fish were checked daily for any clinical signs and mortalities for 10 days. The mortality percentage (MR%) was recorded for each group until the trial ended. MR% and RPS (Relative percent of survival) are calculated as follows:

MR% = (Number of fish mortalities ÷ Total population number) x 100.

RPS% *=* (1– (mortality% in the treated group ÷ mortality% in the control group)) *×*100 (Amend 1981).

**Table 2:** Experimental design and *O. niloticus* groups

Fish groups		Feed additives (SP)	Antibiotic treatment (FF)	No fish/treatment
**1st Trial**	**G1**	×	×	60
	**G2**	×	√	60
	**G3**	√	×	60
	**G4**	√	√	60
**Fish groups**		**Treatment**		**No fish/group**
**2nd Trial**	**G1+**	Fish fed on a basal diet and challenged with *A. hydrophila*		20
	**G2+**	Fish previously fed on a basal diet with FF for 10 days, challenged with *A. hydrophila* and continuously fed on FF for 10 days after the challenge (as therapeutic)		20
	**G3+**	Fish previously fed on SP for 38 days (as prevention), challenged with *A. hydrophila* and continuously fed on SP for 10 days after the challenge		20
	**G4+**	Fish previously fed on SP (38 days) + FF (10 days) (as prevention), challenged with *A. hydrophila*, and continuously fed on SP + FF for 10 days after the challenge		20

SP: Spirulina platensis; FF: florfenicol; Groups; G1; control, G2; fed on FF, G3; fed onSP, G4; fed onSP + FF

### Hematological analysis

Hematocrit (Ht) is the fraction of blood volume occupied by RBCs and is determined by the micro-hematocrit technique (Musuka 2009). A hemocytometer was used to count the RBCs (red blood cells) and WBCs (white blood cells) in the blood that had been diluted 1:200 in Natt & Herrick’s (1951) solution (Bogado et al. 2010). Hemoglobin (Hb) levels were estimated by the cyanomethemoglobin method (Grant 2015). MCV (Mean corpuscular volume), MCH (mean corpuscular hemoglobin), and MCHC (mean corpuscular hemoglobin concentration) were evaluated as stated by the methods of (Briggs and Bain 2012): MCV (fL)= (PCV/RBCs) × 10; MCH (pg)= (Hb, g/100 ml/RBCs) × 10; MCHC (g/dl) = (Hb, g/100 ml/PCV) × 100.

### Biochemical parameters and oxidative stress-related indices

The levels of liver enzymes activity, including ALT (alanine transaminase), ALP (alkaline phosphatase), and AST (aspartate transaminase), were estimated using assay kits from Spectrum, Egypt as stated by Bradley et al. (1972) and Thomas et al. (2005). Creatinine (Diamond Diagnostics Co., Egypt) was measured according to the method of Junge et al. (2004). The levels of antioxidant enzymes were measured using kits from Bio-diagnostic Co., Egypt. SOD (Superoxide dismutase) was measured according to the method of Peskin and Winterbourne (2000). CAT (Catalase) activity was estimated from the reduction in the concentration of H2O2 (Aebi 1984). GPx (Glutathione peroxidase) activity was estimated following Moin (1986).

### Immunological parameters

The phagocytic activity of macrophages was measured according to the methods of Kawahara et al. (1991). The macrophages number was counted to estimate the phagocytic index using the following equations: PA (phagocytic activity) = macrophages engulfing yeast / total count of macrophages × 100(Demers and Bayne 1997); and PI (phagocytic index) = the number of yeast cells phagocytized /number of phagocytic cells.IL10, IL6, and TNF-α were measured using an ELISA plate reader (Table 3) as described by Somade et al. (2019).

**Table 3:** Fish-specified ELISA Kits (Sunlong Biotech Co., LTD, China)

Parameter	Cat No.	Sensitivity	^*^Product Spec
**IL10**	SL0059FI	0.5pg/ml	96T
**IL6**	SL0044FI	0.3pg/ml	96T
**TNF-α**	SL0055FI	0.5pg/ml	96T

^*^Specificity: No significant cross-reactivity or interference between fish parameters and analogs was observed. IL10: interleukin 10, IL6: interleukin 6, TNF-α: tumor necrosis factor α

### Histopathological examination

At the end of the 1st trial, two fish from the control group and five from each of the other groups were randomly sampled for histopathological examination. The abdomen was dissected after the fish were deeply anesthetized with 40% ethyl alcohol and sacrificed via spinal cord transection, then tissue samples from the spleen, kidney, and liver were obtained and fixed in 10% formalin for 24 h, then relocated to 70% ethanol till preparation. The samples were processed in a histoprocessor (TP 1020, Leica Biosystems, Germany). Xylene and a graded ethanol series were used to dry the tissues. The embedded and blocked tissues were sectioned into 5 μm-thick paraffin blocks using a microtome (Leica RM 2125) and stained with H&E later and microphotographed with a digital camera (Leica EC3) connected to a light microscope (Leica DM 5000). Another set of tissues was randomly sampled after the bacterial challenge.

### Statistical analysis

The data were tested using one-way ANOVA (SPSS® version 22, SPSS Inc., IL, USA). Once a treatment effect was found to be significant, a Duncan post-hoc test was utilized to compare the means (Duncan 1955). Treatment effects were quantified at a significance level (P < 0.05). The histopathological changes have been tabulated to assess the frequency and severity of these changes.

## Results

### Dietary SP improves the hematological parameters

Changes in blood indices (RBCs, Hb, Ht, and WBCs) are presented in (Table 4). *O. niloticus* fed on SP (G3 and G4) had significantly higher values of blood indices, indicating an enhanced health status in comparison with the control group, while the FF group (G2) didn’t show any significant differences from the control except for Ht, which displayed a significant decrease.

**Table 4:** Hematological parameters of experimental fish

Items	G1	G2	G3	G4
**RBCs ×10** ^**6**^ **/µL**	3.65^**b**^ ± 0.09	3.23^b^ ± 0.06	4.24^**a**^ ± 0.03	4.15^**a**^ ± 0.2
**Hb g/dl**	12.8^**b**^ ± 0.25	11.25^**b**^ ± 0.26	15.4^**a**^ ± 0.12	14.55^**a**^ ± 0.83
**Ht%**	33.2^**b**^ ± 0.8	29.4^**c**^ ± 0.6	38.6^**a**^ ± 0.26	35.3^**ab**^ ± 1.8
**WBCs** **×10** ^**3**^ **/µL**	31.77^**c**^ ± 0.99	32.55^**c**^ ± 0.5	40^**b**^ ± 1.2	51.6^**a**^ ± 1.4
**MCV fL**	90.96 ± 0.05	91.02 ± 0.17	91.04 ± 0.03	90.99 ± 0.06
**MCH pg**	35.07 ± 0.18	34.83 ± 0.16	36.32 ± 0.03	35.35 ± 0.33
**MCHC g/dl**	38.55 ± 0.17	38.27 ± 0.1	39.9 ± 0.04	38.85 ± 0.39

Values are means ± SE (n = 9/group). Different letters in the same row are significantly different at p ≤ 0.05

### Dietary SP improves the impaired tissue function and structure disrupted by FF

**Fig. 1:**
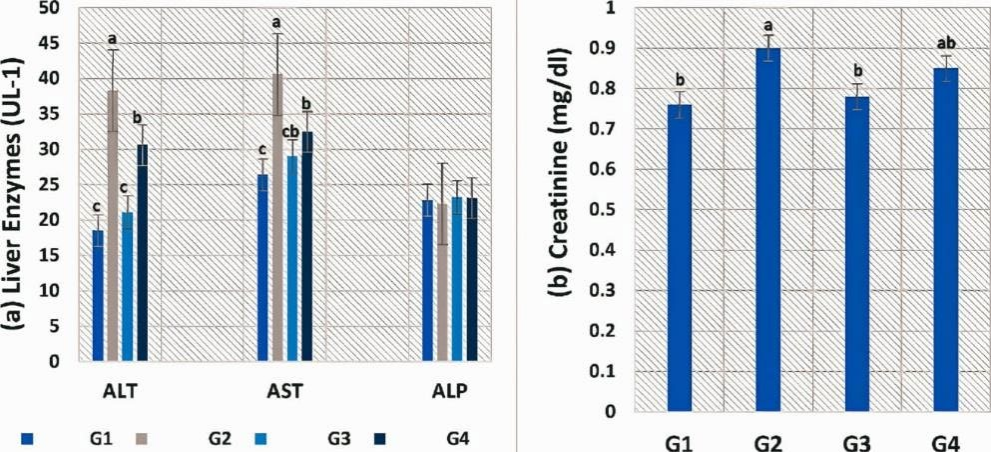
**Serum biochemical parameters of experimental*O. niloticus*groups**, (**a**) Serum liver enzymes (ALT, AST, and ALP) and (**b**) creatinine levels. Values are means; error bars indicate SE (n = 9/group). Different letters above the bars indicate significant differences between treatments (p ≤ 0.05)

In Fig. 1, the treatment with FF had significantly impacted hepatic and renal tissues, as values of liver enzymes (AST, ALT) and creatinine were significantly elevated in G2 compared to other groups. The values of the biochemical indices decreased in fish-fed SP alone or combined with FF, and fish-fed SP alone (G3) showed the best result. However, no variations in the values of these parameters were detected between the control and SP groups. ALP demonstrated insignificant changes between all groups.

**Fig. 2:**
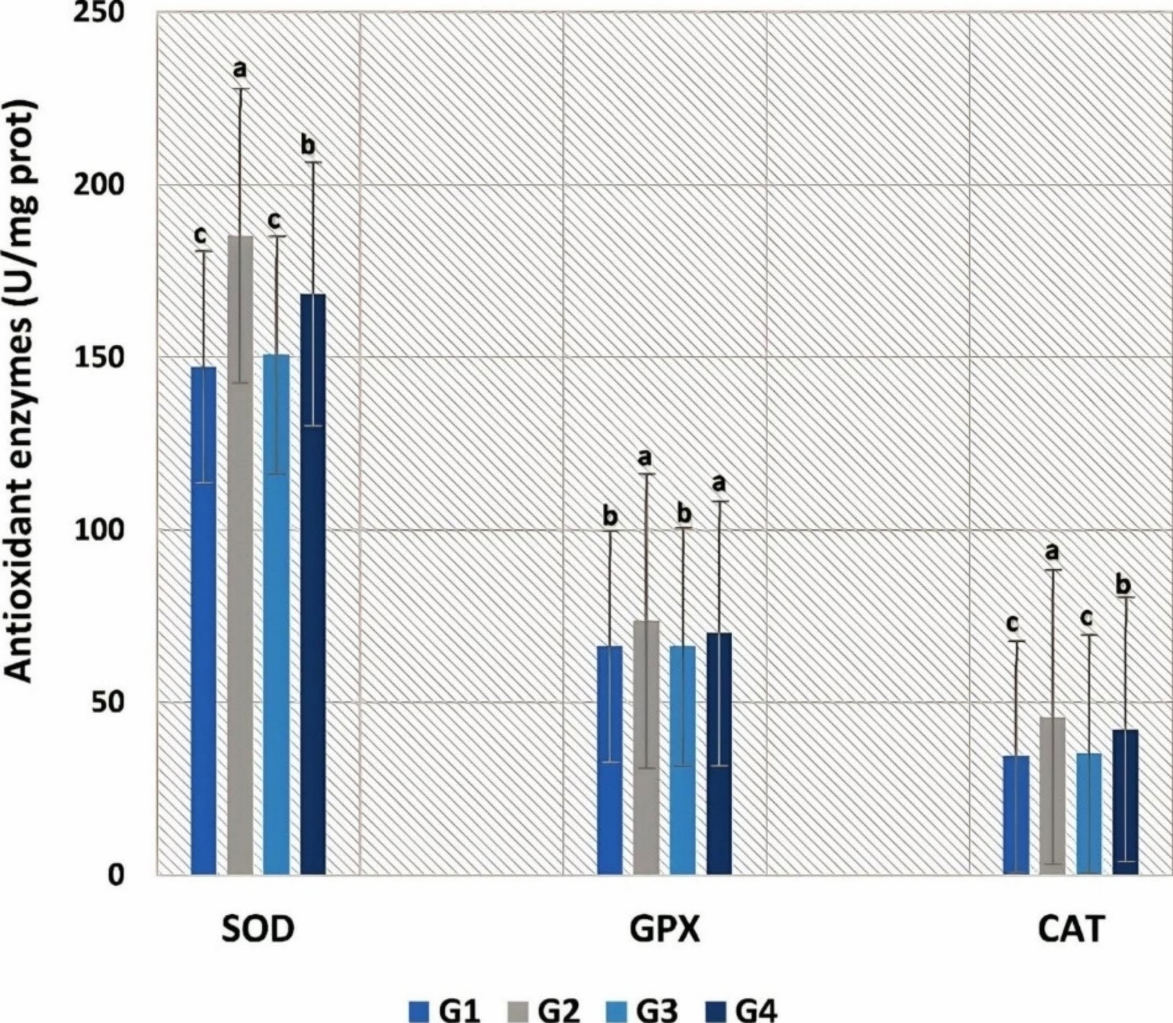
**Serum antioxidant enzymes (SOD, GPx, and CAT) of experimental*O. niloticus*groups**, Values are means; error bars indicate SE (n = 9/group). Different letters above the bars indicate significant differences between treatments (p ≤ 0.05)

Feeding on FF resulted in oxidative stress (Fig. 2), which was expressed by an increase in antioxidant enzymes (SOD, GPx, and CAT) activities in *O. niloticus* (G2). SP could ameliorate such impacts, as G4 (SP + FF) showed lower improvement towards control values, while no significant differences were found between G3 and control groups.

**Fig. 3:**
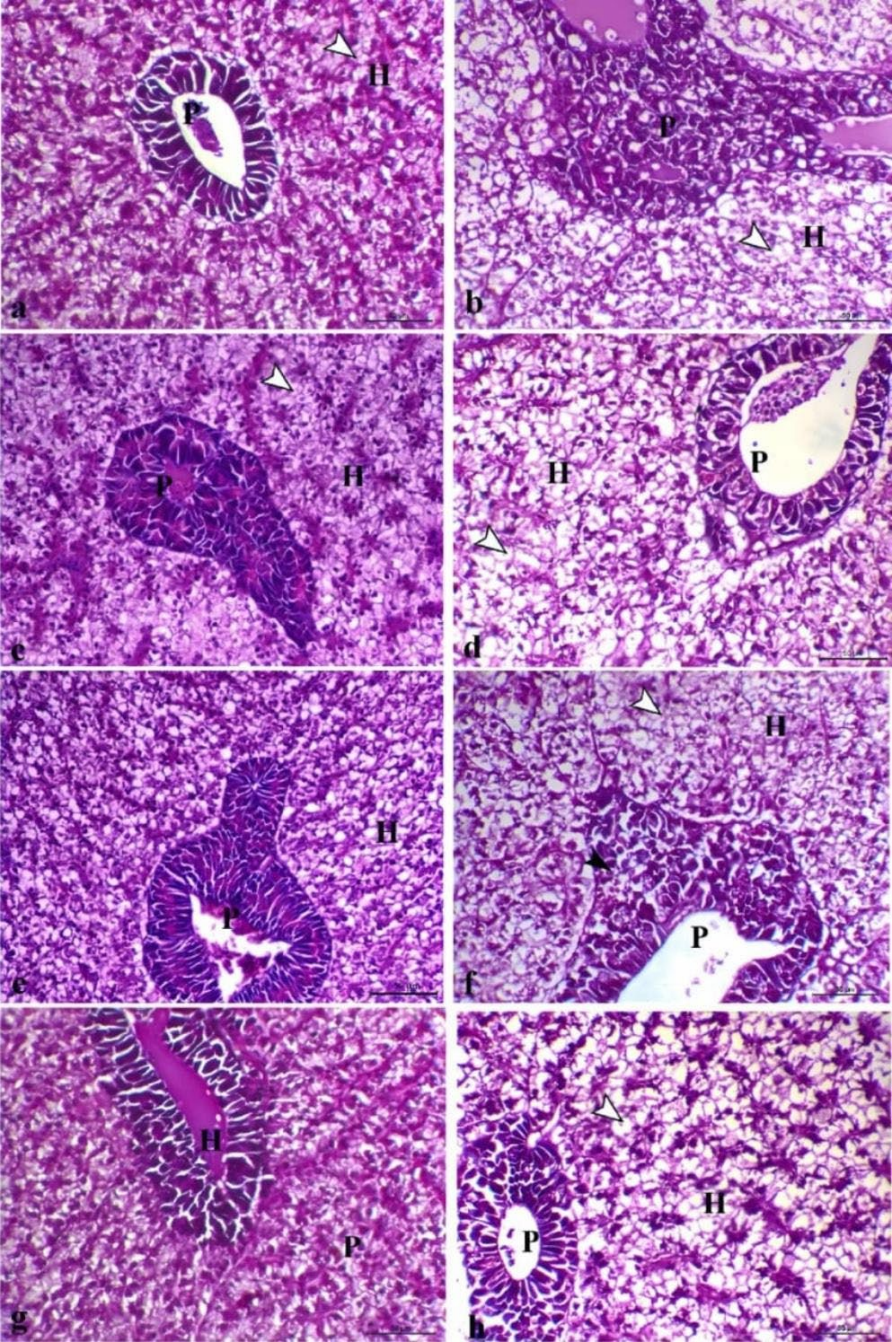
**Photomicrograph of liver sections of different groups, (a)** G1 showing normal hepatocytes and normal pancreatic portion, **(b)** G1 + showing marked degenerative changes representing with severe vacuolation of hepatocytes (arrowhead), and degeneration and necrosis of the pancreatic cells, **(c)** G2 showing normal hepatic tissues with mild hepatic vacuolation consistent with glycogen storage (arrowhead) and normal pancreatic portions, **(d)** G2 + showing decrease vacuolar degenerative changes within both hepatocytes (arrowhead) and pancreatic cells, **(e)** G3 showing normal hepatic portion and normal pancreatic portion, **(f)** G3 + showing decrease vacuolar degenerative changes within the hepatocytes (white arrowhead) and moderate degenerative changes with pancreatic cells associated with mild hemosiderosis (black arrowhead), **(g)** G4 showing normal hepatocytes and normal pancreatic portions, **(h)** G4 + showing marked decrease the degenerative changes within both hepatic and pancreatic cells (arrowhead indicates moderate degree of hepatic vacuolar changes), H letter refers to hepatocytes and P letter refers pancreatic portion, H&E,X200, bar = 50 μm

Histopathological investigation of the liver revealed normal hepatocytes and normal pancreatic portions in G1, G3, and G4 (Fig. 3a, 3e, and 3g respectively). However, in G2, tissue damage was detected in the form of mild hepatic vacuolation consistent with glycogen storage (Fig. 3c). The histopathological changes of *O. niloticus* groups were evaluated quantitively (Table 5) and the bacterial infection was reported to cause vacuolar degenerative changes within the hepatocyte and degenerative changes within pancreatic cells, which varied from marked changes representing severe vacuolation of hepatocytes and degeneration and necrosis of the pancreatic cells in G1+ (control infected) (Fig. 3b), decreased in FF; G2+ (Fig. 3d), decreased vacuolar degenerative changes within the hepatocytes and moderate degenerative changes with pancreatic cells in the fish group supplemented with SP; G3+(Fig. 3f), This disturbed tissue damage was partially restored in G4+ (FF + SP) group, which showed a marked decrease in the degenerative changes within both hepatic and pancreatic cells (Fig. 3h).

**Table 5:** The histopathological changes of *O. niloticus* groups as evaluated quantitively on ^*^a seven-point scale

Organ	G1	G1+	G2	G2+	G3	G3+	G4	G4+
kidney								
**DE**	-	2.21 ± 0.15^b^	-	0.53 ± 0.11^e^	-	0.89 ± 0.01^d^	^−^	0.23 ± 0.06^f^
**V**	-	2.14 ± 0.08^b^	-	0.42 ± 0.08^e^	-	-	^−^	0.19 ± 0.12^f^
**EI**	-	2.75 ± 0.11^b^	-	-	-	0.76 ± 0.05^d^	-	
**IF**	-	1.83 ± 0.07^c^	-	0.96 ± 0.03^d^	-	-	-	
**Liver**								
**GV**	-	3.47 ± 0.15^a^	0.98 ± 0.19^d^	0.35 ± 0.07^e^	-	-		0.15 ± 0.07^f^
**VD**	-	2.35 ± 0.04^b^	-	0.46 ± 0.17^e^	-	0.52 ± 0.20^e^		0.23 ± 0.04^f^
**PD**	-	1.31 ± 0.07^c^	-	0.38 ± 0.13^e^	-	1.91 ± 0.20^c^		0.20 ± 0.18^f^
**PN**	-	1.23 ± 0.09^c^	-	-	-	-	-	
**Spleen**								
**↑ LC, ↑MMC**	-	3.69 ± 0.02^a^	2.96 ± 0.08^b^	2.57 ± 0.02^b^	2.82 ± 0.09^b^	2.23 ± 0.03^b^	2.75 ± 0.43^b^	3.01 ± 0.02^b^
**NLC NMMC**	-	3.44 ± 0.24^a^	-	-	-	-	-	
**LH**	-	-	-	-	-	-	2.15 ± 0.52^b^	-

DE: Degeneration of renal tubular epithelium; V: Vacuolation; EI: granular eosinophilic cell infiltration; IF: interstitial fibrosis; GV: vacuolation with Glycogen storage; VD: Vacuolar degeneration; PD: pancreatic degeneration; PN: pancreatic necrosis; LC: lymphoid cells; MMC: melanomacrophage cells; NLC: necrosis of lymphoid cells; NMMC: necrosis of melanomacrophage cells; LH: lymphoid hyperplasia. Values are the mean of five observations for each organ of the respective group except for the control group, there are two observations of each organ. *Seven-point scale: -: no change, a: severe, b: marked, c: Moderate, d: mild, e: decrease, and f: marked decrease. G1; control -ve, G1+; control infected with *A. hydrophila*, G2; fed on FF, G2+; fed on FF and infected with *A. hydrophila*, G3; fed on SP, G3+; fed on SP and infected with *A. hydrophila*, G4; fed on SP + FF, G4+; fed on SP + FF and infected with *A. hydrophila*

**Fig. 4:**
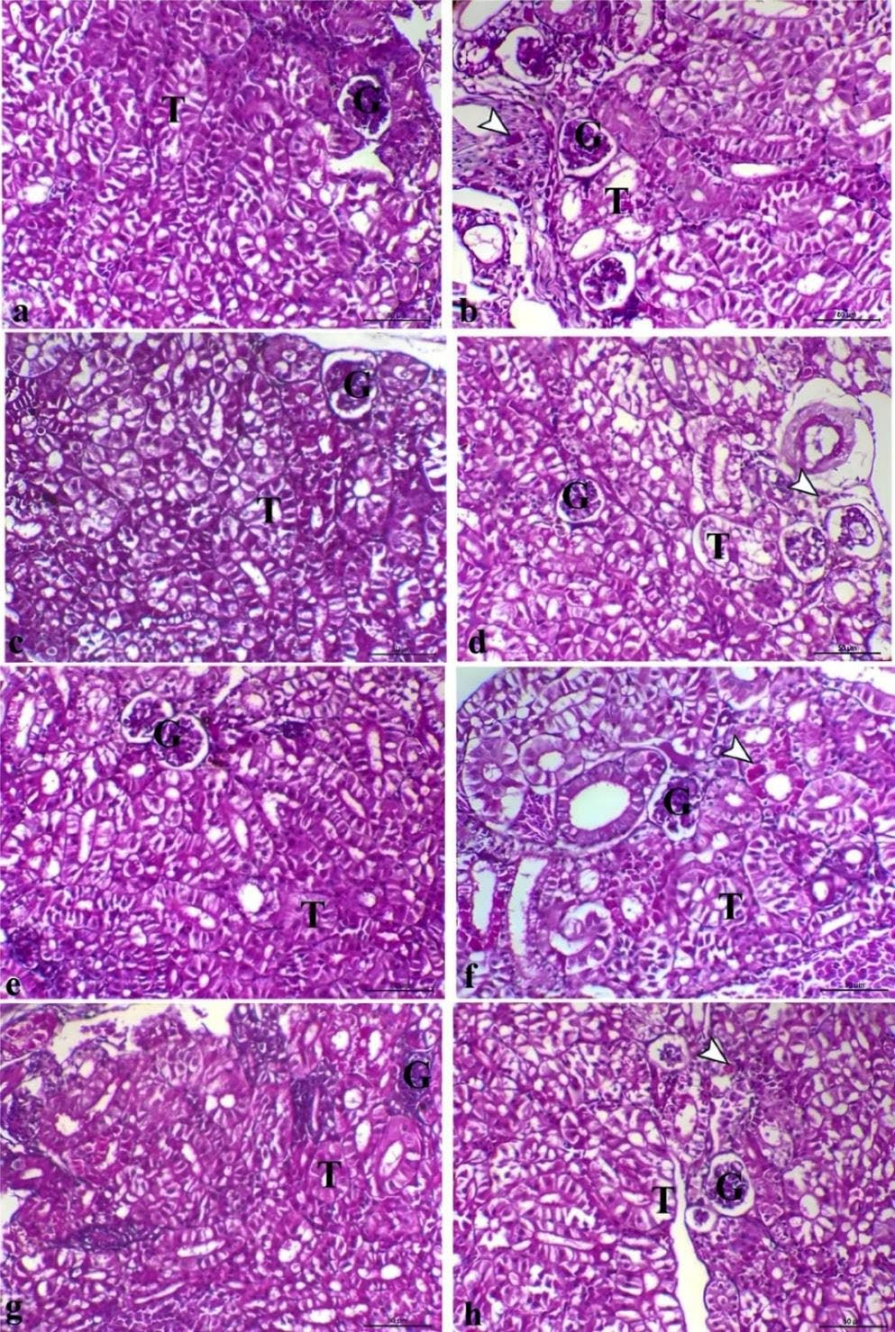
**Photomicrograph of kidney sections of different groups, (a)** G1 showing normal renal glomeruli and tubules, **(b)** G1 + showing marked vacuolar degenerative changes within the renal tubular epithelium, interstitial fibrosis, and granular eosinophilic cells infiltration (arrowhead), **(c)** G2 showing normal renal glomerular and tubular tissues, **(d)** G2 + showing decrease the vacuolar degenerative changes within the renal tubular epithelium and mild granular eosinophilic cells infiltration (arrowhead), **(e)** G3 showing normal renal glomerular and tubular structures, **(f)** G3 + showing focal eosinophilic granular degenerative changes within the renal tubular epithelium, **(g)** G4 showing normal renal glomerular and tubular structures, **(h)** G4 + showing marked decrease the vacuolar degenerative changes within the renal tubular epithelium, G and T letters indicate glomerulus and tubule respectively, H&E, X200, bar = 50 μm

Like to liver, the kidney of G1 (Fig. 4a), G2 (Fig. 4c), and G3 (Fig. 4e) in addition to the G4 (Fig. 4g) had normal renal glomerular and tubular tissues. Bacterial infection impacted the renal tissues causing vacuolar degenerative changes within the renal tubular epithelium, and granular eosinophilic cells infiltration, which varied from marked changes in the G1+ (Fig. 4b), decreased changes and mild granular eosinophilic cells infiltration in G2+ (Fig. 4d), focal eosinophilic granular degenerative changes in G3+ (Fig. 4f). Once more, kidney of G4 + showed marked decrease the vacuolar degenerative changes which indicate enhancement with tubular regeneration (Fig. 4h).

**Fig. 5:**
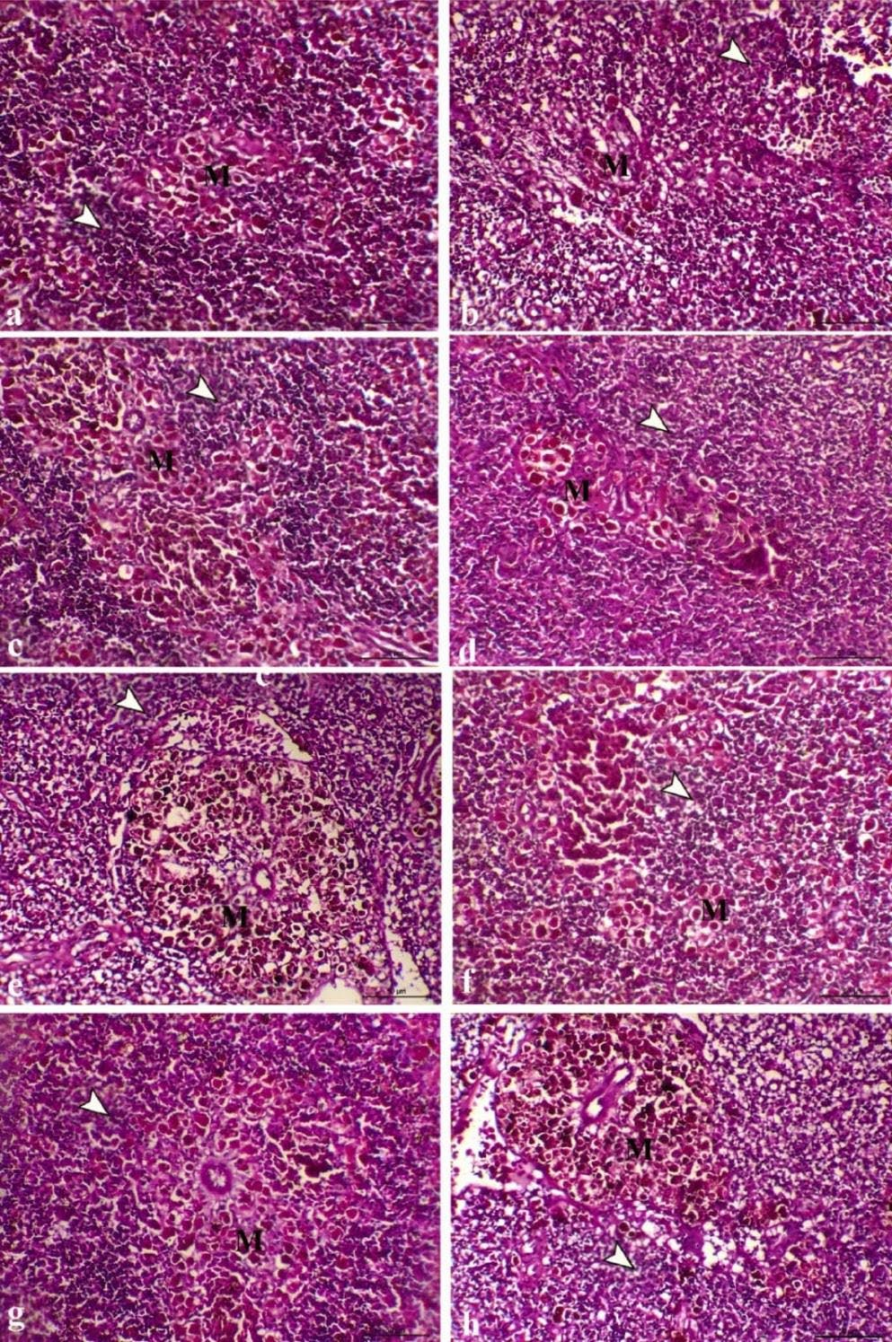
**Photomicrograph of spleen sections of different groups, (a)** G1 showing normal white pulp consisting of lymphoid cells (arrow) and melanomacrophage cells, **(b)** G1 + showing marked lymphoid necrosis of both lymphoid cells (arrow) and melanomacrophage cells, **(c)** G2 showing a marked increase of lymphoid cells (arrow) and melanomacrophage cells within the white pulp, **(d)** G2 + showing an increase of both lymphoid cells (arrow) and melanomacrophage cells within the white pulp, **(e)** G3 showing a marked increase of lymphoid cells (arrow) and melanomacrophage cells within the white pulp, **(F)** G3 + showing an increase of both lymphoid cells (arrow) and melanomacrophage cells within the white pulp, **(G)** G4 showing marked lymphoid cells hyperplasia (arrow) and melanomacrophage cells within the white pulp,**(h)** G4 + showing a marked increase of both lymphoid cells (arrow) and melanomacrophage cells, M letter refers to melanomacrophage cells, H&E, X200, bar = 50 μm

Histopathological investigation of the spleen showed a normal white pulp consisting of lymphoid cells and melanomacrophage cells in the control group (Fig. 5a), while FF treatment and SP supplementation alone or in combination caused a marked increase in lymphoid cells and melanomacrophage cells within the white pulp (Fig. 5c, 5e and 5g respectively). Bacterial infection caused marked lymphoid necrosis of both lymphoid cells and melanomacrophage cells in G1+ (Fig. 5b), while in treated groups (G2+, G3+, G4+) caused an increase of both lymphoid cells and melanomacrophage cells within the white pulp (Fig. 5d, 5f and 5h respectively).

### Dietary SP improves the immunomodulatory activities of FF

**Fig. 6:**
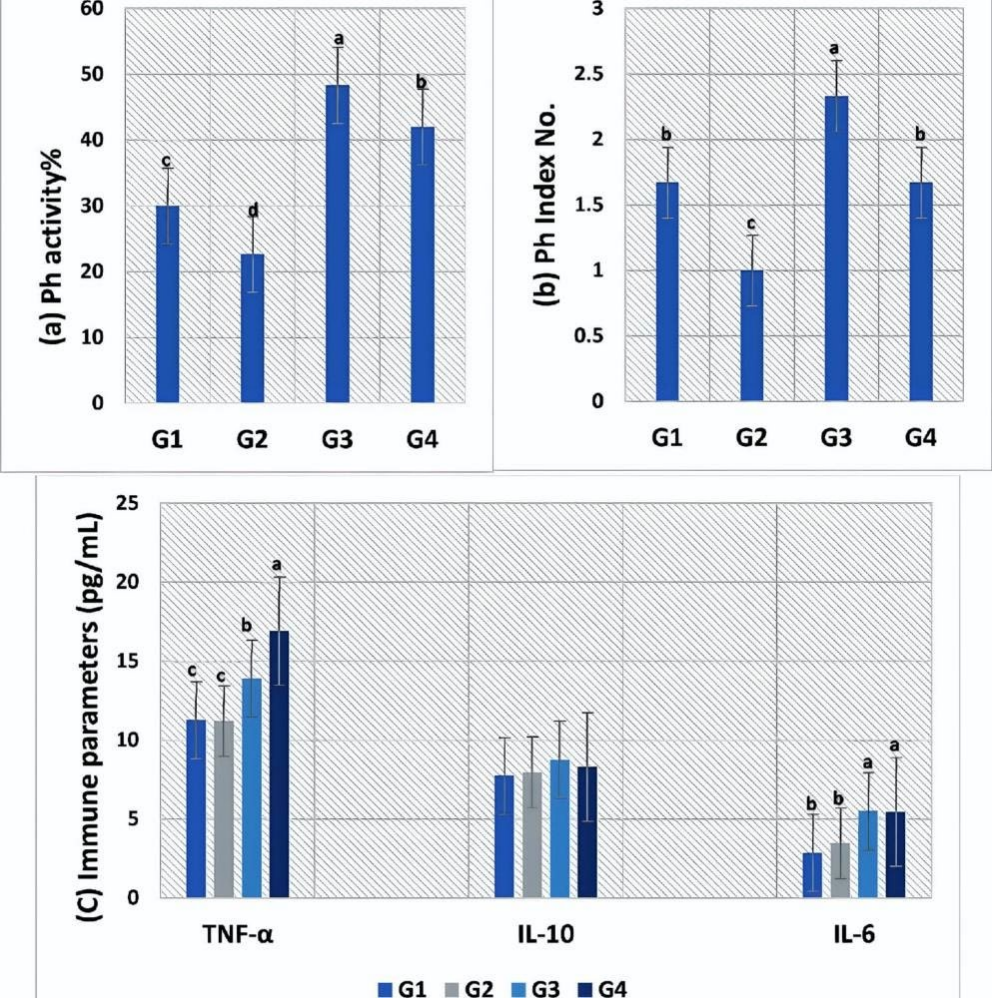
**Immune parameters of experimental*O. niloticus***, (**a**) Phagocytic activity, (**b**) phagocytic index, (**c**) TNF-α, IL-10, IL-6 levels. Values are means; error bars, indicate SE (n = 9/group). Different letters above bars indicate significant differences between treatments (p ≤ 0.05)

Figure (6) discusses the effects of dietary SP and/or FF on immune parameters. Fish that received SP (G3 and G4) were significantly higher in phagocytic activity and index (Fig. 6a and b) compared to other groups. FF treatment showed a decrease in the activity of phagocytic cells. The results revealed a marked increase in the serum levels of pro-inflammatory cytokines IL-6 and TNF-α in the fish-fed SP diet in contrast to those fed on the FF diet, which displayed insignificant differences from the control (Fig. 6c). All groups had no significant changes in IL10 levels.

### Dietary SP improves feed intake, survival rate, and relative percent of survival

In Table 6, G1, G3, and G4 were actively feeding 100% during the 1st feeding trial. Feed intake% was decreased in G2 to 96.79 ± 1.29%, while bacterial infection during the 2nd trial had impacted the feed intake which reduced to 24.41 ± 2.29, 87.43 ± 2.03, 86.21 ± 3.09% in G1+, G2+, and G3 + respectively. G4 + showed the best improvement in feed intake, which displayed 93.25 ± 1.02%. No mortalities were detected among fish groups in the 1st feeding trial except for two mortalities in G2. MR% was decreased in infected fish that received dietary FF and/or SP, G2+, G3+, and G4+ (30, 35, and 10% respectively), while G1 + recorded higher mortalities (60%). The dietary SP (G3+) achieved RPS of 41.67% which was raised to 83.33% when combined with FF treatment (G4+), whereas, FF treatment alone(G2+) had a low RPS of 50%.

**Table 6:** Feed intake, mortality rate (MR), and relative percent of survival (RPS) in the experimental *O. niloticus*

Items	G1	G1+	G2	G2+	G3	G3+	G4	G4+
**Fish no.**	60	20	60	20	60	20	60	20
**Feed intake%**	100 ± 0.00^a^	24.41 ± 2.29^d^	96.79 ± 1.29^b^	87.43 ± 2.03^c^	100 ± 0.00^a^	86.21 ± 3.09^c^	100 ± 0.00^a^	93.25 ± 1.02^b^
**Fish death**	-	12	2	6	-	7	-	2
**MR%**	0^d^	60^a^	3.33^c^	30^b^	0^d^	35^ab^	0^d^	10^ cd^
**RPS**%	-	-	-	50^b^	-	41.67^c^		83.33^a^

Values are means ± SE (n = 240). Different letters in the same row are significantly different at p ≤ 0.05

## Discussion

FF is a wide-ranging antibiotic used to treat various fish bacterial diseases (Gaikowski et al. 2013). The antioxidant enzymes and antioxidant activity in numerous fish species were reported to be destroyed by FF and drug administration (Limbu et al. 2018; Shiroma et al. 2020; Bardhan et al. 2023). This motivates us to search for a proper neoadjuvant to work synergistically with FF and reduce adverse effects. SP is applied successfully in fish feed as an immunostimulant supplement with the possible impact to diminish oxidative stress (Rosas et al. 2019). There was insufficient data available about the probable role of SP in addressing the impacts of FF on aquatic organisms, therefore the current trial focused on evaluating SP as a natural and eco-friendly feed additive to enhance the antioxidative status and blood biochemical parameters of *O. niloticus* treated with FF.

In the current investigation, the hematological parameters of *O. niloticus* were improved by SP, which shows that SP is a safe supplement for *O. niloticus* diets. This improvement might be attributed to algae’s high iron concentration, which affects erythropoiesis (Khalil et al. 2017). Similar findings showed that supplemental SP dramatically improved blood parameters in *Oncorhynchus mykiss* (Yeganeh et al. 2015). Gabriel et al. (2007) documented that the elevated WBCs counts in the blood were caused by *Cyprinus Carpio’s* fight against atrazine toxicity, which was improved by the SP supplement. In another study, *Oplegnathus fasciatus* which received a diet treated with 5% *Spirulina Pacifica*, had higher PCV values than control fish (Kim et al. 2013).In this investigation, all blood parameters analyzed for *O. niloticus* treated with FF were all within the normal range except for Ht which displayed a significant decrease and these results were compatible with Shiroma et al. (2020).

Long-term antibiotic therapy causes oxidative damage to the liver and kidney (Reda et al. 2016). To evaluate if SP combined with FF could improve these side effects, we examined changes in some liver and kidney damage markers (ALT, AST, ALP, and creatinine) and discovered that SP modulated the FF-induced increase in these markers. The reversal of these indicators by SP to control levels demonstrates that SP may have hepatic and renal protective activity. The amelioratory effects of SP were verified by Sayed and Fawzy (2014). The authors recognized that the abnormal blood biochemical indices caused by diet stress in *Clarius garipinus* including, AST, ALT, ALP, urea, and creatinine were modulated by SP. Karadeniz et al. (2009) and Simsek et al. (2009) observed that SP showed modulatory effects in rats suffering from liver impairment caused by Cd and Pb exposure. SP showed no adverse impacts on the liver function of *O. niloticus*, which may be because of its different antioxidant content, which minimizes cell damage and helps to repair and regenerate damaged cells (Stivala et al. 1996). The histopathological findings of the kidney and liver showed this protective role. Hepatic damage was detected in the FF group in the form of mild hepatic vacuolation consistent with glycogen storage, which demonstrated the depletion of glycogen reserves as well as the hepatotoxic potential of FF. Hepatocyte vacuolation is indicated by increased hepatocyte space and hazy cell margins. Hepatocyte vacuolization is a sign of degeneration that could lead to metabolic dysfunction, which in our study could be associated with FF treatment and bacterial infection. Similar results were obtained by Bardhan et al. (2022b) and this may be attributed to the ROS (reactive oxygen species) overproduction, which damages the cell membrane and leads to ion pump destruction, which is the first step in the process of vacuolar degeneration, resulting in the release of creatinine and liver enzymes (Abdelhadya et al. 2017). This could clarify the reason why serum levels of those markers are so high in the FF group. A significant decrease in histopathological changes in the liver tissues at the bacterial infection concomitant with the treatment with the best improvement in the SP + FF group.

Our results revealed that feeding on FF resulted in oxidative stress, which was expressed by the elevation of GPx, CAT, and SOD activities. Again, the inclusion of SP restored antioxidant enzymes level to normal. This minimizes the severity of oxidative damage to the liver and kidney induced by FF. The 10 days of FF treatment were documented to cause oxidative damage to *O. mykiss* (Elia et al. 2016). The amelioratory effect of SP is possibly attributable to its antioxidant activities that improve CAT and SOD levels in tissues of the kidney, gill, and liver (Wu et al. 2016; Mohanty and Samanta 2018). The antioxidant activities of phenolic acids, β-carotene, tocopherols, minerals, and vitamins found naturally in SP may be responsible for its ROS scavenging abilities (Abdel-Daim et al. 2013).

Treatment with FF and SP in the current study decreased the pathological changes brought on by bacterial infection in all the examined organs, with a clear effect in the SP + FF group. Similarly, silver bream treated with natural additives have improved hepatic tissue structure (Ostaszewska et al. 2008). Oxidative stress induction is partially responsible for the detrimental effects of bacterial infection in the tissues of the host (Tkachenko et al. 2014). Because of its ability to reduce oxidative stress and thus relieve the damaging effects, the SP mode of action has become the magic bullet.

Histopathological investigation revealed that FF caused mild hepatic vacuolation consistent with glycogen storage, and a marked increase in lymphoid cells and melanomacrophage cells within the white pulp of the spleen with no effect on the renal tissues. Because of the low mortality rate (3.33%) shown in the FF group, these alterations are predicted to have negligible therapeutic significance. Similar to our results, Bardhan et al. (2022a) reported cytoplasmic degeneration, glycogen-type vacuolation, nuclear abnormalities, and cellular hypertrophy in the liver of FF-treated *O. niloticus* for 10 days suggesting its hepatotoxic potential. Melanomacrophage cells are pigmented phagocyte aggregations in the spleen and head kidney related to immunity by phagocytosing foreign agents (Steinel and Bolnick 2017). Closely comparable to our results, an upsurge in MMC was documented in *O. niloticus* fed a diet enriched with *Echinacea purpurea* (El-Asely et al. 2012) and in farmed *Dicentrarchus labrax L.* fed a diet supplemented with polyphenol (Magrone et al. 2016).

In the same trend, Straus et al. (2012) found that FF-fed fish showed no signs of renal tubular necrosis, while Bardhan et al. (2022a) documented that the ten days of FF treatment caused renal tubular inflammation, degeneration, and necrosis. The frequency and severity of degenerative changes within the renal tubular epithelium and granular eosinophilic cell infiltration were higher in the control infected group (G1+) in comparison with the treated infected groups (G2+, G3+, G4+), which may result in severe renal failure. G4 + exhibited the best improvement with the regeneration of renal tubules. The observations on the degenerative changes within the renal tubular epithelium proposed that the damage caused by *A. hydrophila* might be inflammatory, ischemic, and obstructive. The permanence of these alterations suggested the probable nephrotoxic impact of *A. hydrophila*, which could lead to tissue necrosis.

To gain a better understanding of the immunostimulatory activity of SP, the immune indices were evaluated. Moreover, SP alone or in combination with FF improved immunity as seen by increased levels of phagocytic activity and index, pro-inflammatory cytokines (IL-6), and TNF-𝛼. Again, the SP + FF group had the strongest immune-stimulatory effects, demonstrating further facet of synergism between SP and FF. When compared to the control fish, FF alone triggered a reduction in the activity of phagocytic cells and a low phagocytic index and insignificantly affected the immune parameters (IL-6, IL10, and TNF-𝛼). As stated by (Lundén et al. 2002), rainbow trout given FF (10 mg/ kg BW) daily for ten days had a considerably reduced blood phagocytic index. When Reda et al. (2013) fed *O. niloticus* FF for 3 months at a minimal dose (5 mg/ kg BW/ day) and they investigated the changes in phagocytic activity, immunoglobulin levels, and plasma lysozyme levels but were unable to identify any. Despite a minor effect on the phagocytic activity being recorded, FF insignificantly affected the immunological responses of *O. mykiss* at 20 mg kg/ BW (Lundén et al. 1999). These confusing results could be the consequence of different administration methods, dosages, temperatures, and fish species. The FF-induced reduction of the total number of blood neutrophils, which is the reason for the macrophages’ increased phagocytic activity, may be the cause of the decrease in the phagocytic index. Our results are corroborated by those of El-Sheekh et al. (2010), who revealed that SP could improve immune responses by modulating macrophage function, phagocytic activity, and IL-1 production. Thus, to maintain better immune responses, FF should be added to fish diets combined with SP rather than alone (as a prophylactic treatment or as a growth promotor if permitted according to country laws and rules).

Regardless of the immune-suppressive impacts of FF, when combined with SP, the highest immune response and disease resistance were attained. This was evident in the lower mortalities in the challenged fish fed SP + FF compared to those fed separately. The increased survival in challenged fish-fed SP versus the control infected group provided further evidence of these better immune responses to SP. In addition to SP’s immune-stimulating effects, *O. niloticu’s* survival following bacterial infection can also be a result of its anti-inflammatory abilities, as demonstrated by an increase in the levels of the cytokine IL6, which is a strong pro-inflammatory cytokine. Our observations were confirmed by those of Cao et al. (2018), who noticed that even at minimum dosages (3.38 g/kg of feed), adding SP to the diet of juvenile Gibel carp significantly reduced MR% following 7 days of *A. hydrophila* challenge as compared to control. In light of the improvement in immunological response and growth, the authors recommended these substances as feed additives and immunostimulants against some infections. Our results revealed that dietary SP enhances RPS when followed by FF treatment after the bacterial challenge. Fish-fed combined SP and FF had the greatest antibacterial effect, indicating another type of synergism between SP and FF against microorganisms. This can be explained by combining the antibacterial activity of SP with that of FF. After an experimental challenge with *A. hydrophilia*, Watanuki et al. 2006 assessed the changes in the bacterial numbers in fish organs treated with SP. They reported that the bacterial cell number was relatively low in the kidney and liver of carp fed-SP compared to the control, indicating improved resistance against *A. hydrophilia* infection.

Several European countries have outlawed the use of antibiotics as a feed supplement to stimulate growth due to the high potential of antimicrobial residues in the tissues of animals and the development of resistant bacteria. These resistant bacteria can transmit to humans, producing significant health problems because the majority of them are lethal and cannot be treated (Kesarcodi-Watson et al. 2008). This prohibition would most certainly reduce the growth rate while increasing production costs. As a result, researchers need to seek substitutes that imitate the favorable effect of antibiotics as growth enhancers. Some of these options, such as SP, probiotics, and acidifiers, may be able to replace antibiotics in fish feed (Khalil et al., 2017; Reda et al. 2016). However, enhancements in immunity and health status as a result of these alternatives have frequently been inconsistent and variable. So, it is better to combine these alternatives with an appropriate amount of antibiotics. We applied the same method here, combining dietary SP with FF, revealing an enhancement in the immune response and antimicrobial benefits.

## Conclusion

As far as we know, this is the first investigation to demonstrate the synergistic impact of SP and FF when added to *O. niloticus* diets. In conclusion, exposure to FF resulted in oxidative damage in *O. niloticus* but did not affect the hematological parameters. Our findings thus imply that even at therapeutic doses, FF antibiotics may be detrimental to *O. niloticus*. The combined SP and FF decreased oxidative stress and reduced liver, kidney, and spleen tissue damage induced by FF and/or bacterial infection. Our results contribute to the description of possible risks associated with this antibiotic in aquatic ecosystems and might be utilized to progress further management plans to diminish the use of these chemicals in aquaculture to minimize their effects on fish health. Furthermore, this trial promotes the use of alternate additives, such as immunostimulants (SP) in combination with an appropriate amount of antibiotics, to fight diseases and it increases awareness about the overuse of antibiotics to reduce environmental hazards and bacterial resistance.

## Data Availability

The authors confirm that the data supporting the findings of this study are available within the manuscript, figures, and tables.
